# Heat therapy in individuals at risk for Alzheimer’s disease—methods for a randomized controlled trial

**DOI:** 10.3389/fneur.2026.1736108

**Published:** 2026-01-23

**Authors:** Paige C. Geiger, Jenae S. Pennington, Paul J. Kueck, Casey S. John, Hana D. Mayfield, Riley E. Kemna, Jeffrey Burns, Eric Vidoni, Robyn Honea, Yanming Li, Jonathan Mahnken, Jill K. Morris

**Affiliations:** 1 Department of Cell Biology and Physiology, University of Kansas Medical Center, Kansas City, KS, United States; 2 University of Kansas Diabetes Institute, University of Kansas Medical Center, Kansas City, KS, United States; 3 Department of Neurology, University of Kansas Medical Center, Kansas City, KS, United States; 4 University of Kansas Alzheimer’s Disease Research Center, Fairway, KS, United States; 5 Department of Biostatistics and Data Science, University of Kansas Medical Center, Kansas City, KS, United States

**Keywords:** Alzheimer’s disease, dementia, glucose metabolism, heat therapy, metabolic syndrome

## Abstract

Heat therapy (HT) has been shown to improve peripheral blood glucose regulation in some populations, yet its effects on brain glucose metabolism remain largely unexplored. The chronic benefits of HT may arise in part from upregulation of heat-shock proteins (HSPs). These proteins play a crucial role in the stress response and modulate diverse processes such as proteostasis and cell signaling pathways, including that of insulin signaling. Understanding the impact of HT on both peripheral and central glucose metabolism, including the effects of varying temperatures, is essential for elucidating potential mechanisms underlying its brain benefits. The Feasibility of Improving Glycemia to prevent Alzheimer’s Disease (FIGHT-AD) study is a randomized controlled trial that aims to investigate changes in blood and brain glucose regulation following 10 weeks of HT. Specifically, we will examine the peripheral biomarker responses to warm and hot HT and assess how these responses relate to brain metabolic changes in both treatment groups. This trial will be the first to quantify the effect of HT on cerebral glucose metabolism in individuals at metabolic risk for Alzheimer’s Disease (AD). The FIGHT-AD trial will provide critical data to inform the design of future clinical trials targeting metabolic and brain health through HT.

**Clinical trial registration:**

clinicaltrials.gov, identifier NCT06023407.

## Highlights

- This study is designed to evaluate the metabolic effects of a 10-week Heat therapy (HT) intervention in older adults.
- Main whole-body outcomes are HbA1c, insulin sensitivity index, and continuous glucose monitoring.
- Brain glucose metabolism measured by FDG-PET imaging will be evaluated as a secondary outcome.
- Vascular measures such as blood pressure and effects on AD-relevant blood biomarkers will also be assessed.

## 1 Introduction

Alzheimer’s Disease (AD) is the leading form of dementia and it is estimated that AD could affect approximately 15 million people by 2050 (1). The incidence of prediabetes and diabetes increase substantially with age, which is important because there is accumulating evidence that metabolic and vascular diseases increase risk for Alzheimer’s Disease (AD).

Heat Therapy (HT), primarily via hot water immersion that raises core body temperature, has been independently shown to improve risk factors for cardiometabolic disease. Long term HT (8–10 weeks) results in improvements in blood glucose regulation, insulin resistance, inflammation, and blood pressure in both young, healthy individuals and in obese populations (2–5). Importantly, improvements in these modifiable cardiometabolic outcomes could lower risk for AD (6–10). It is well established that fasting glucose levels increase with aging, and that impaired glucose metabolism is a risk factor for AD (11).

Prior work suggests that peripheral glucose levels have an inverse relationship with brain glucose metabolism (12). Brain hypometabolism, a marker of reduced energy metabolism, is also a reliable and early diagnostic biomarker of AD (12, 13). In cognitively healthy older adults, peripheral hyperglycemia is associated with decreased cerebral glucose metabolism in several brain regions on Flurodeoxyglucose positron emission tomography (FDG-PET) including the precuneus, posterior cingulate, and parietal regions (14–17). These regions are core components of a set of connections called the Default Mode Network, which are regions that exhibit some of the highest metabolic rates in the brain (18, 19), hypo-metabolism in AD (12, 13), and are among the first to accumulate amyloid (20). This suggests that improving peripheral hyperglycemia may be particularly important for brain health in individuals with elevated glucose levels.

Here, we describe the clinical trial protocol of the Feasibility of Improving Glycemia to prevent AD (FIGHT-AD) study. This trial compares the effects of hot and warm water immersion on blood and brain glucose metabolism in cognitively healthy older adults with metabolic syndrome following a 10-week intervention. Hot water immersion has a more robust effect on core temperature compared with other whole body heating modalities (i.e., traditional sauna and far-infrared sauna approaches) (21), potentially affording the greatest physiological responses in our study population. Individuals will undergo up to 30 sessions of hot (40.5 °C, *n* = 30) or warm (36 °C, *n* = 30) water immersion to assess the effect of temperature on the changes in blood and brain glucose metabolism (pre to post). We hypothesize that 10 weeks of thrice weekly HT will lower hemoglobin A1c (HbA1c) values and improve insulin sensitivity index outcomes. Additionally, we hypothesize that individuals will improve (increase) global cerebral glucose metabolism following 10 weeks of HT. We will also explore effects on other outcome measures such as blood pressure, blood AD biomarkers, and brain blood flow.

## 2 Materials and equipment

Whole body and regional fat mass, fat-free mass, percent body fat, and bone mineral density will be assessed using a dual energy x-ray absorptiometry scan (DEXA) scan (GE Lunar iDXA).

Heart rate and blood pressure will be captured with an automatic blood pressure monitor (Connex® Spot Monitor, Welch Allyn, Inc). An IV catheter will be placed for blood collection and assessment of a Glucose Tolerance Test. Participants will be instructed to consume a 75 g beverage (TRUTOL® 75, Fisher Diagnostics) at the start of the test. Flurodeoxyglucose (FDG) is administered with FDG-PET, performed with a GE Discovery ST-16 PET/CT scanner.

Magnetic Resonance Imaging (MRI) will be performed with a Siemens 3.0 Tesla Skyra scanner. G6 Pro Continuous Glucose Monitoring (CGM) will be utilized (Dexcom, San Diego, CA). Core temperature will be assessed and recorded using eCelsius Performance Pills and ePerformance Manager software (BodyCap, Hérouville-Saint-Clair, Normandy, France). Urine specific gravity to confirm euhydration will be determined with a refractometer (Fisherbrand™ Handheld Analog Clinical Refractometer, Thermo Fisher Scientific, Waltham, MA). Hot water and thermoneutral immersion will be performed in two commercial hot tubs (Marquis Rendezvous and Freeflow Tristar Spa). Whole blood will be analyzed at baseline for HbA1c using the DCA Vantage Analyzer (Siemens, Tarrytown, NY) and for lipid analysis using the Cholestech LDX Lipid Profile Test Cassette (Abbott, San Diego, CA). TNF, IL-6: Cytokine 4-Plex A (C4PA) Advantage PLUS, Neuro 2 Plex B assay (N2PB) will be measured using the Simoa HD-X (Quanterix, Billerica, MA) and phosphorylated tau 217 and amyloid beta 42 on the Lumipulse G1200 (Fujirebio).

## 3 Methods

### 3.1 Trial design

**Figure 1:**
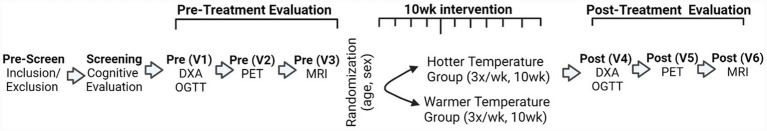
Study flow for FIGHT-AD. Pre-screening by the KU ADRC recruitment division will result in pass-offs to the study team. Participants will be consented prior to cognitive evaluation to screen out cognitive impairment. Pre-treatment baseline evaluations will be performed at 3 in-person visits (OGTT, MRI, PET). CGM will also be placed at visit 3. After completing all 3 pre-treatment evaluation visits and meeting inclusion criteria, participants will be randomized to intervention groups (Hot water or Warm water). Participants will attend intervention or thermoneutral visits with study team at treatment evaluations and complete the study.

FIGHT-AD is a two-arm, prospectively enrolling randomized controlled trial that compares the effects of thrice weekly HT in water immersion for 10 consecutive weeks on glucose metabolism in warm (*n* = 30) and hot (*n* = 30) water. Participants will complete telephone cognitive testing and clinical evaluation to screen for any cognitive impairment. After screening, informed consent will be obtained and participants will be enrolled into the study. Cognitively healthy older adults who meet the criteria for metabolic syndrome will be enrolled and complete (1) a fasting blood draw; (2) an Oral glucose tolerance test (OGTT); and (3) CGM, MRI and an FGD-PET scan. Assessments will be repeated following 10 weeks of HT. Participants will undergo 30 sessions of HT in 40.5 °C water or 36 °C water to assess the effect of temperature on changes in blood and brain glucose metabolism. The flow of the study is illustrated in Figure 1.

#### 3.1.1 Trial registration and outcome measures

The initial IRB approval for the FIGHT-AD study occurred on March 3, 2023. The trial was registered on clinicaltrials.gov on August 28, 2023 (NCT06023407). Upon completion of the trial, results will be made available on clinicaltrials.gov. Upon primary analysis and publication of the study outcomes, data will be made publicly available to other researchers. The primary outcomes are changes in glycated hemoglobin (HbA1c) and insulin sensitivity index (ISI). The secondary outcome measure is change in cerebral glucose metabolism, quantified as [18F] FDG global standardized uptake value ratio (SUVR) assessed pre- vs. post-HT. Exploratory outcome measures include the effects of HT on blood pressure, inflammation (CRP, TNF, IL-6, JNK and IKK) and AD biomarkers (Amyloid/Tau/Neurodegeneration measures), and neuroimaging measures of vascular function.

### 3.2 Recruitment and participants and ethics approval

Participants are recruited and prescreened through the University of Kansas Alzheimer’s Disease Research Center. All study visits occur at the University of Kansas Medical Center. A trained study staff member obtains informed consent from all participants through a signed consent form on the day of their first visit. This study was approved by the Institutional Review Board (IRB) at the University of Kansas Medical Center (IRB: STUDY00147285). Any modifications to the research protocol, should they be made, will be reviewed and approved by the IRB. All procedures are performed in accordance with the Declaration of Helsinki. All reasonable efforts are made to ensure data and participant privacy and confidentiality. Upon completion of the study, we will provide a letter to participants informing them of the study findings and where they can access the resulting publication.

Participants complete an initial screening process to determine their eligibility. Eligible participants are 65 years and older and have been evaluated to be cognitively healthy (Clinical Dementia Rating (CDR) = 0) using a remote CDR and single clinician diagnosis (22). Individuals must be on stable medication doses, and female participants must be post-menopausal to participate. Furthermore, participants must have an existing diagnosis of either prediabetes or type 2 diabetes (T2D). If a participant does not have prediabetes or T2D, participants must have 2 out of the 3 following: BMI > 30, Hypertension, or Dyslipidemia. Individuals with type 2 diabetes treated with non-insulin pharmacologic agents were eligible for inclusion provided medication doses were stable for at least one month prior to enrollment; insulin-dependent diabetes mellitus was excluded. A complete list of inclusion and exclusion criteria are provided in Table 1.

**Table 1:** Inclusion and exclusion criteria used during pre-screening process.

Inclusion criteria	Exclusion criteria
Age 65 and older Stable medication doses (>1 month) Post-menopausal Clinical Dementia Rating (CDR) of 0 History of or current metabolic impairment (i.e., metabolic syndrome, pre-diabetes, Type 2 Diabetes, etc)	Excluded from or unable to complete an MRI scan. MRI compatible pacemakers will require cardiologist clearance prior to enrolling. ACSM Risk score stratification of “High” unless cleared by a physician prior to participation. Myocardial infarction or symptoms of coronary artery disease in the last 2 years. History of or current diagnosis of disorders with the potential to impair cognition (i.e., AD, Parkinson’s disease, stroke (defined as clinical episode w/ neuroimaging evidence in appropriate area to explain symptoms)). Insulin-dependent Diabetes Mellitus. Clinically significant chronic disease such as cancer, HIV, or acquired immunodeficiency syndrome. Clinically significant depressive symptoms that may impair cognition, use of psychoactive and investigational medications, and significant visual or auditory impairment. Orthopedic complications that would preclude individuals from safely entering a hot tub. Current or significant history of lower body wounds or diabetic ulcers Untreated hypothyroidism or diseases associated with heat intolerance (i.e., Graves disease, etc.). Contraindication for temperature pill ingestion (i.e., inflammatory bowel disease, diverticulitis or related). Participants may participate in other observational studies but cannot participate in other studies in which they receive an intervention.

MRI, Brain magnetic resonance imaging; ACSM, American College of Sports Medicine; AD, Alzheimer disease; HIV, Human immunodeficiency virus.

### 3.3 Procedures

Overall study flow is shown in Figure 1 and list of procedures at each study visit is given in Table 2. First, participants are screened by phone and basic demographic information is collected. The American College of Sports Medicine Participation Health Screening was used to assess risk stratification. Participants who were characterized as “High Risk” were excluded unless a physician clearance was provided. Once deemed eligible, all subsequent screening procedures and in-person study visits are completed over the course of about 14 weeks. Participants are administered a neuropsychometric exam (National Alzheimer’s Coordinating Center Uniform Data Set telephone cognitive battery; T-Cog) by a trained psychometrician, as well as a telephone diagnostic assessment (Telephone CDR; T-CDR) by a trained clinician. Following participant consent, medical history and medication use information are collected via patient report. Pre-treatment assessment also includes a dual energy x-ray absorptiometry scan (DEXA) and OGTT. At the following visit, participants are administered FDG prior to the PET, to measure brain glucose metabolism. CGMs will be utilized to assess 24 h continuous glucose measures in a daily living environment. Each participant also completes a MRI scan.

**Table 2:** Procedures administered at each telephone or in-person study visit.

Assessment	Pre-screen	Visit 1	Visit 2	Visit 3	Intervention (10-weeks)	Visit 4	Visit 5	Visit 6
Demographics	X							
Medical history	X	X				X		
Medications	X	X				X		
Telephone CDR	X							
Telephone cognitive test	X							
Anthropometrics/Vitals		X			X	X		
DEXA		X				X		
Blood draws					X (Wk 4)	X		
Oral glucose tolerance test				X	X (after final treatment)			
Continuous glucose monitoring (7 days following visit)		X				X		
FDG-PET scan			X				X	
MRI				X				X
Randomization				X				
Heat or thermoneutral sessions					X			

CDR, Clinical dementia rating; DEXA, Dual energy X-ray absorptiometry; FDG-PET, (18)F-fluorodeoxyglucose positron emission tomography; MRI, Magnetic resonance imaging.

Following successful completion of baseline assessments, participants are randomized to receive treatment in hot or warm water temperature group. An allocation table was created by the unblinded study statistician working with the study team. Randomization is performed using the REDCap randomization module (23). Individuals are randomized 1:1 to intervention group according to the allocation table accounting for sex and age to ensure approximately equal representation among HT groups. At each subsequent study visit, participants complete 45 min of HT. Assessment of DEXA, GTT, FDG-PET, CGM and MRI are repeated after 10 weeks HT. Study staff who oversee therapy sessions will be unblinded to study group. All outcome assessors (psychometricians, nursing staff, neuroimaging technicians, etc) are blinded to participant intervention group. Study data is stored using REDCap software in a secure study database.

#### 3.3.1 Visits 1 & 4

##### 3.3.1.1 Dual energy X-ray absorption scan

The first and fourth assessment is scheduled in the morning following an overnight fast, with the latter (post-intervention visit) being scheduled at most 72 h after their last HT session. Following informed consent, information regarding demographics, and anthropometric data is collected. At each visit participants change into lightweight hospital gowns and are instructed to remove all metal objects (i.e., jewelry, hairpins, etc.) that may interfere with accuracy of the measurement. Participants remain in the supine position throughout the DEXA scan (GE Lunar iDXA). This provides data for whole body and regional fat mass, fat-free mass, percent body fat, and bone mineral density. The scanner is calibrated each day prior to use according to manufacturer guidelines (22).

##### 3.3.1.2 Oral glucose tolerance test

Following the DEXA scan, heart rate and blood pressure are measured after 5 min of seated rest. Heart rate (HR) and Blood pressure (BP) are captured with an automatic blood pressure monitor (Connex® Spot Monitor, Welch Allyn, Inc). An IV catheter is placed for blood collection throughout the study visit and the baseline draw is collected. Participants are instructed to consume the 75 g beverage (TRUTOL® 75, Fisher Diagnostics) within 5 min and OGTT begins once the entire beverage has been consumed. Blood Draws 2, 3, 4 are collected at minutes 30, 60 and 120, respectively. Blood processing procedures are described in section 3.4.

#### 3.3.2 Visits 2 & 5 FDG-PET scan

At the FDG-PET scan, participants arrive after having fasted approximately 8 h. Participants receive a single IV bolus of FDG approximately 30 min prior to the start of the scan. FDG-PET are performed with a GE Discovery ST-16 PET/CT scanner. The FDG-PET scan includes six, five-minute frames that are attenuation corrected and reconstructed into a single FDG-PET image. FDG-PET images will be analyzed using custom software written for Statistical Parametric Mapping (SPM12). For each participant, the FDG-PET images from both the pre- and post-HT condition will be co-registered to the participant’s pre-anatomical MRI image. *A priori* individualized, native space region of interest masks will be generated from the Computational Anatomy Toolbox (CAT12) for SPM. The FDG-PET images will be SUVR of the cerebellum.

#### 3.3.3 Visits 3 & 6 brain magnetic resonance imaging (MRI) and continuous glucose monitoring (CGM)

Participants complete an MRI safety screening upon arrival to Hoglund Biomedical Imaging Center. Scans are performed with a Siemens 3.0 Tesla Skyra scanner. High-resolution T1 weighted images (MP-RAGE; 1 × 1 × 1 mm voxels; TR = 2,300, TE = 2.98, TI = 900, FOV 240 × 256, 1 mm slice thickness, flip angle 9 degrees) are acquired for detailed anatomical assessment and co-registration of the PET scans. In addition to structural measures, we also obtain measures of fluid attenuated inversion recovery (FLAIR; 0.9375 × 0.9375 × 5.0 mm voxels, TR = 9,000, TE = 91.0, FOV = 256 × 256, flip angle 150 degrees) cerebral blood flow (pcASL; pseudocontinuous, 3.125 × 3.125 × 2.5 mm voxels, TR = 4,300, TE = 22.42, FOV 672 × 672, flip angle 120 degrees), and cerebral oxygen extraction fraction (T2 -relaxation-under-spin-tagging; TRUST; 3.4375 × 3.4375x5mm voxels, TR = 3,000, TE = 7.1, FOV 64 × 64, flip angle 90degrees). G6 Pro Continuous Glucose Monitoring (Dexcom, San Diego, CA) will be utilized and worn for 7–10 days pre- and post-intervention.

### 3.4 Heat therapy and thermoneutral control sessions

Participants will be scheduled for 30 sessions over 10 weeks of either hot water immersion (heat group) or warm water immersion (thermoneutral control) group. Specific methods for each group are described below.

#### 3.4.1 Core body temperature measurement

Intestinal temperature is used to estimate body core temperature in all participants and measured via a telemetric pill BodyCAP USA (eCelcius). The pill travels through the gastrointestinal tract and transmits temperature to a receiving device outside of the subject’s body. Body core temperature may also be measured using an oral thermometer. The pill typically passes through an individual’s system within a few days. The pill is first administered upon arrival to the CTSU and 30 min prior to water immersion. Once core body temperature is detected, it is measured at 10 min intervals throughout the duration of the intervention.

Upon arriving at CTSU, study staff will check to see if core temperature senor is still reading from previous visit. If signal still present, no new sensor is provided. When no signal is present, a new sensor is ingested and the participant rests for 30 min. The signal is checked again prior to immersion. If the sensor has no or bad signal, oral temperature will be recorded during the visit.

#### 3.4.2 Water immersion (heat therapy) group

Participants will be immersed up to the shoulder in a 40.5 °C water therapy bath until core body temperature (Tc) reaches 38.5 °C, ~30 min, as previously established with updates via personal communication (3). Participants will then remain in the water bath submerged to waist level to maintain core temperature between 38.5 and 39.0 °C for another 15 min (total of 45 min). Following heat therapy, Participants will exit the hot tub and be monitored for another 10 min, or until core temperature falls below 38.5 °C. Participants are asked to provide a urine sample to check urine specific gravity to confirm euhydration via urine specific gravity < 1.02 using a refractometer (Fisherbrand™ Handheld Analog Clinical Refractometer, Thermo Fisher Scientific, Waltham, MA). If urine specific gravity is > 1.02, subjects are asked to consume 5 mL kg − 1 of water prior to entering the hot tub (3).

Signs and symptoms of heat related illness are also continuously monitored by study staff. HR and BP measurements are taken every 10 min during the study visit. If heart rate increases > 60 beats/min above resting or increases > 20 beats/min within a 5 min time period, participants are moved to a seated position if they were previously fully submerged or removed from the hot tub if they were already sitting up. If body temperature reaches 39.5 °C or the participants experience symptoms of heat related illness, we will begin a Rapid Cooling Protocol: (1) Immediately remove subject from the hot tub; (2) Place ice packs in subject’s groin and axillae (armpits); (3) Mist subject constantly using a spray bottle filled with tepid (15 °C) water; (4) Place a large fan to circulate warm room air directly at the subject; (5) Continuously monitor body temperature, blood pressure, and heart rate for 20 min. Participants are encouraged to drink water ad libitum during the session. Body weight is measured using a scale before and immediately after all sessions. Participants that do not drink enough water to compensate for sweat loss (body weight loss > 1%) will drink additional fluids to make up this difference prior to leaving the study visit.

#### 3.4.3 Warm water immersion (thermoneutral control)

Because hydrostatic effects of water immersion may affect physiology, we include a comparison group comprised of individuals exposed to warm water immersion (thermoneutral condition). We follow procedures identical to the heat therapy protocol, except that the warm water therapy bath will be 36 °C. Prior work has shown that these conditions maintain core temperature within 0.2 °C of resting (3). This will ensure that both groups are exposed to conditions that are equivalent in terms of any hydrostatic effects, engagement, and patient experience.

### 3.5 Participant engagement

Participants are encouraged to attend all assigned sessions. Participants receive renumeration upon completion of the baseline study visits, as well as renumeration for the intervention visits. In addition, upon enrollment participants receive a small welcome package containing items relevant to the intervention that may improve their experience (i.e., towel, insulated water bottle, wet/dry bag, etc).

### 3.6 Blood processing

At the baseline timepoint of Visit 1, we collect whole blood for apolipoprotein E4 (*APOE4*) genotyping, which is immediately frozen in polypropylene cryovials at -80C. Whole blood is also analyzed at baseline for HbA1c using the DCA Vantage Analyzer (Siemens, Tarrytown, NY). Additionally, 2 mL of whole blood is collected in a 2 mL lithium heparin tube for lipid analysis using the Cholestech LDX Lipid Profile Test Cassette (Abbott, San Diego, CA). Finally, we collect 10 mL of blood into EDTA plasma tubes at all timepoints of the OGTT. At the baseline timepoint, we generate both platelet-rich plasma (PRP) and platelet-poor plasma (PPP). For subsequent time points at 30, 60, and 120 min, only PRP is generated. PRP is prepared by centrifuging whole blood at 1500 x g for 10 min at 4 °C, followed by careful removal of plasma. PPP requires an additional centrifugation step at 1700 x g for 15 min at 4 °C. Plasma will be frozen and analyzed at the end of the study for various biomarkers using immunoassays for the specific proteins of interest: TNF, IL-6: Cytokine 4-Plex A (C4PA) Advantage PLUS, Neuro 2 Plex B assay (N2PB) on the Simoa HD-X (Quanterix, Billerica, MA) and phosphorylated tau 217 and amyloid beta 42 on the Lumipulse G1200 (Fujirebio). As an exploratory outcome, plasma heat shock protein 70 (HSP70) will be quantified to assess heat therapy–related activation of the heat shock response.

### 3.7 Statistical analysis plan and power

We will conduct an analysis to assess changes in blood glucose regulation in response to 10 weeks of HT of either thermoneutral or hot water immersion. The primary outcome measures are change in HbA1c and ISI from pre- to post-heat therapy. A secondary outcome measure is change in global FDG SUVR from the pre- to post-intervention.

The primary statistical analyses will contrast change scores in HbA1c and ISI following 10 weeks of HT between the temperature groups (warm vs. hot) using two-sample paired t-tests. We will conduct residual analyses to assess the appropriateness of this methodology to evaluate assumptions of common variance, normally distributed errors, etc. In the event any of the measures do not conform to these foundational assumptions, the nonparametric analogs will be used instead, such as the Wilcoxon rank sum test of the within-subject baseline vs. post-treatment stimulated change score (difference) measures. These measures will follow the intent-to-treat principals. Though a randomized design, we will also construct exploratory analyses that incorporate other adjustments using ordinary least squares (OLS) regression on the change scores as the outcomes, also with residual analysis and modified approach if indicated. This will allow for model adjustment to further examine these relationships.

One further relationship will involve combining both HT groups in a single model. Finally, we will model change score outcomes as functions of the various other change score measures. This approach will enable us to observe how changes in HbA1c and ISI are driven by heat therapy-influenced changes in other factors, such as circulating biomarkers. We will conduct similar residual analyses appropriate to the regression models utilized to ensure appropriate inferences are drawn. While descriptive and exploratory in nature, we anticipate that this approach will illuminate and/or confirm pre-conceived metabolic processes that are related to the HT response, and that it will help identify likely reasons for prior HT trial successes and failures through mechanistic discovery.

For Aim 1, we calculated the sample sizes needed for achieving different levels of power (80–95%), with respect to different effect sizes. The effect size here is defined as pre- and post- intervention change in ISI. The effect size 0.42 was given from Ely et al., who tested a similar heat therapy intervention (3x per day for 10 weeks) in a younger, obese population (3). Given the different patient population, use of this effect size could be a potential limitation. Effect sizes 0.24 and 0.15 were computed by subtracting 1 and 1.5 times of the pooled standard error (SE) of 0.18 (see Table 1 in Ely et al.) from 0.42. The effect size 0.1 is very conservative and corresponds to subtracting 1.8 times the SE from 0.42. That effect size will require 51 samples to achieve 80% of power. We expect to potentially have 10% attrition and an extra 5% missing data in each group. With these considerations, our planned sample size of 30 per group would provide more than 85% power to detect change in our primary Aim 1 outcome, even withstanding possible attrition and missing data.

### 3.8 Safety and data monitoring

The HT presents risks associated with heat exposure, including fatigue, light-headedness, muscle cramps, dehydration, and neurological detriments (i.e., heat stroke). However, core temperature is monitored throughout the HT and a rapid cooling protocol is in place if core temperature exceeds 39.5C°. The study team continually monitors for adverse events during the entirety of study participation. MRI scanning is not generally associated with any health risks. Individuals with claustrophobia or non-compatible implanted devices are excluded from the study. The FDG-PET scan requires radiation exposure, though the amount of radiation and health risk associated therein are minimal. Remaining safety concerns are minimal, and include typical risks associated with blood draw (site swelling, pain, bruising) and cognitive testing.

The FIGHT-AD study uses a Data Safety Monitoring Committee comprised of field experts external to the University of Kansas Medical Center. This Committee meets every 6 months to review study recruitment and adverse events.

## 4 Anticipated results

HT has emerged as a promising, non-pharmacologic intervention that is being investigated as an intervention to reduce the risk of AD. We are focusing on its effects on blood and brain glucose metabolism, and other factors such as proteostasis, inflammation, and vascular function —factors linked to AD neuropathology. Our recent pilot study provided evidence for safety and feasibility of a 4-week heat therapy intervention protocol in cognitively healthy older adult populations (24). The 10-week FIGHT-AD study will add to our understanding of the impact of HT on whole body metabolic function and brain health.

For the proposed study, individuals will undergo up to 30 sessions of hot (40.5 °C, *n* = 30) or warm (36 °C, *n* = 30) water immersion to assess the effect of temperature on the changes in blood and brain glucose metabolism (pre to post). We hypothesize that 10 weeks of HT will lower HbA1c values and improve insulin sensitivity index outcomes. Additionally, we hypothesize that individuals will improve (increase) global cerebral glucose metabolism following 10 weeks of HT. We will also explore effects on other outcome measures such as blood pressure, blood AD biomarkers, and brain blood flow.

## 5 Discussion

The pathological hallmarks of AD have been described for over 100 years and include the presence of amyloid plaques and neurofibrillary tangles. These protein deposits are thought to occur as a result of failed protein homeostasis, or proteostasis (25–27). Proteostasis ensures protein quality control and functional capacity through a variety of mechanisms, including proper protein folding by chaperones. Age dependent declines, in protein chaperones known as HSPs could leave neuronal and non-neuronal cells open to proteotoxic insults and increase the risk for the development of neurodegenerative diseases, including AD (28, 29). HSPs specifically facilitate many processes known to benefit cellular health. These include the folding of newly synthesized proteins, the refolding of damaged proteins, targeted degradation of non-functional proteins and organelles, prevention of oxidative damage, import/export of proteins into/out of the mitochondria, and intracellular signaling (including insulin signaling) (30–34). These mechanisms highlight their importance in protecting against AD development.

The HSR is a conserved cellular mechanism that upregulates heat HSPs to mitigate cellular stress and maintain cellular function, with links to longevity (35, 36). HSPs play a critical role in maintaining cellular function through the regulation of protein folding and degradation. Studies suggest that induction, transcription, and translation of these cytoprotective HSPs decline in chronic metabolic and neurodegenerative diseases (37–39). Conversely, induction of HSPs results in ample metabolic benefit in animal models of obesity and metabolic diseases (37, 40–45). Mechanistically, this link of HSP’s to human metabolic disease and diseases of aging and cognitive decline makes sense. Activation of the transcription factor heat shock factor 1 (HSF1) initiates the heat shock response in the cytoplasm (46). HSF1 is activated in response to elevated temperature, oxidant exposure, metals and other conditions associated with protein misfolding, perhaps through an internal temperature sensor mechanism that is not yet fully understood (47, 48). Importantly, HSF1 is decreased in brains from AD animal models and also decreases with increasing severity of neuropathology (Braak staging) in AD brains from human subjects (49). This suggests that the HSR is a viable target for improving brain health and addressing neurodegenerative conditions like AD.

Proteins regulating HSF1, such as glycogen synthase kinase 3 (GSK-3), are altered with brain aging. Elevated GSK-3 activity promotes tau pathology, impairs insulin signaling, and worsens glucose metabolism, contributing to AD risk (50). Importantly, GSK-3 is a stress kinase that can impair insulin signaling and glucose metabolism, and inhibition of GSK-3 is well known to improve whole body insulin resistance and glucose metabolism, and is being investigated as a potential AD therapy (50–53). HT enhances HSP expression, improves glucose regulation, and reduces fasting glucose and insulin resistance in preclinical mechanistic studies (41, 54). We hypothesize that HSP upregulation via HT will improve insulin sensitivity and brain glucose uptake in older adults at metabolic risk for AD, potentially impacting systemic inflammation, biomarkers, and brain function.

The benefits of HT are attributed to the induction of molecular chaperones, such as heat shock proteins (HSPs), which support protein refolding, mitochondrial function, and oxidative damage prevention (32, 33, 35, 55, 56). A pilot study by our group demonstrated that HT reduced inflammatory markers in fibromyalgia patients. Given inflammation’s role in AD (AD), we will explore HT’s effects on biomarkers of proteostasis (e.g., HSPs), inflammation (CRP, TNFα, IL-6, IL-10), and AD neuropathology (A/T/N measures). MRI will assess intervention-related changes in brain metabolism, blood flow, and brain oxygen extraction.

### 5.1 Limitations and considerations

As mentioned, pilot work has provided evidence for safety and feasibility of HT in cognitively healthy older adult populations. This study will expand upon that work by providing characterization of important physiological endpoints that have been linked to brain health. However, some limitations remain. Although an intermediate time point (4 weeks) is included, we do not measure acute changes. In addition, we may be underpowered to detect sex differences, should any occur. Nonetheless, this study will provide important information that can be used to power future studies focused on such outcomes. Finally, it remains unclear as to whether this intervention is safe and feasible in cognitively impaired populations. Further studies regarding these limitations are warranted.

## 6 Conclusion

In conclusion, HT has emerged as a promising, non-pharmacologic intervention that is being investigated as an intervention to reduce the risk of AD. Here we describe the methods for our study assessing the effects of HT on blood and brain glucose metabolism, and other factors such as proteostasis, inflammation, and vascular function —factors linked to AD neuropathology. AD, a progressive and incurable disease, is projected to affect 15 million people by 2050. Given these associations, incorporating HT into preventative strategies for AD could offer a novel approach to mitigate its onset, aligning with the urgent need for alternative treatments to address this global health challenge (1, 57).
